# Self‐reported disease symptoms of stone quarry workers exposed to silica dust in Ghana

**DOI:** 10.1002/hsr2.189

**Published:** 2020-10-05

**Authors:** Dzifa Francis Ahadzi, Abdul‐Rahaman Afitiri, Bernard Ekumah, Verona Kanatey, Abdullah Afedzi

**Affiliations:** ^1^ Center for Occupational Safety and Health (COSH) Health and Safety Group Africa (HESAG Africa) Cape Coast Ghana; ^2^ Department of Environmental Science, School of Biological Sciences College of Agriculture and Natural Sciences, University of Cape Coast Cape Coast Ghana

**Keywords:** disease symptoms, Ghana, occupational, self‐reported, silica dust, stone quarry

## Abstract

**Background and aims:**

Understanding the importance of using personal protective equipment (PPE) and the influence of work‐post (working distance to main dust source—crusher) in stone quarries is vital for designing tailored interventions in minimizing workers' exposure to silica dust. Nonetheless, studies on silica dust and disease symptoms in Ghana are nascent. This study assessed how work‐post and use of required PPE jointly influence exposure to silica dust and disease symptoms in Ghana.

**Methods:**

Generalized linear models (complementary log‐log regression) were fitted to cross‐sectional survey data of 524 stone quarry workers in Ghana to assess the joint effect of work‐post and PPE usage on self‐reported disease symptoms while controlling for relevant compositional and contextual factors.

**Results:**

Stone quarry workers who work between 1‐100 m and beyond 100 m from the crusher with the required PPE were 90% and 87% respectively less likely to report eye irritation compared with their counterparts who work between 1 and 100 m from the crusher without the required PPE. Individuals who work between 1‐100 m and beyond 100 m from the crusher with the required PPE were 94% and 95% respectively less likely to report breathing difficulty compared with the reference group. Workers who work between 1‐100 m and beyond 100 m from the crusher with the required PPE were 97% and 99% respectively less likely to report coughing compared with the reference group. Workers who work between 1‐100 m and beyond 100 m from the crusher with the required PPE were 93% and 97% respectively less likely to report common cold compared with their counterparts who work between 1 and 100 m from the crusher without the required PPE.

**Conclusion:**

There are adverse health implications for people who work in silica dust polluted environments, suggesting the need for a national safety and health policy to target them.

## 1 INTRODUCTION

One of the major causes of occupational disease in the world is exposure to respirable crystalline silica (RCS). Globally, millions of industrial workers are exposed to silica dust. In China, an estimated 23 million workers are exposed to silica dust while in India, about 10 million workers are exposed.^1^ Similarly, in the United States, 2.3 million workers are estimated to be exposed to silica dust with 1.85 million of them in the construction sector.^2^ Recent studies have shown increased morbidity and mortality among silica‐exposed workers.^1^ In Britain, nearly 800 people die every year from lung cancer and about 7000 people suffer from lung cancer annually in Europe as a result of inhaling RCS at work.^1^ Mwaiselage et al,^3^ found in their study that chronic cough and work‐related shortness of breath are associated with cumulative silica dust exposure.

Crystalline silica refers to the chemical compound silicon dioxide (SiO_2_), which occurs in a crystalline or non‐crystalline (amorphous) form. The amorphous form of silica is less hazardous compared with the crystalline form.^4^ Crystalline silica is one of the most abundant minerals in the earth's crust.^4^, ^5^, ^6^ It is present in almost all types of rock, sand, clay, shale, gravel as well as products such as bricks, tiles, concrete, and some plastic composites.^7^, ^8^, ^9^, ^10^ The main forms of crystalline silica are quartz, cristobalite, and tridimite. Silicon dioxide is most prevalent in the mineral quartz. The toxic form of crystalline silica is less than 5 μm in size and is called RCS.^1^ RCS is one of the oldest and most dangerous workplace hazards in the world and is a known human carcinogen.^5^, ^11^, ^12^ High concentrations can be inhaled unknowingly due to its very fine form. When inhaled, RCS causes serious damage to the lung when it reaches the extremities of the lung and penetrate deep into it in sufficient quantity.^2^, ^6^, ^11^, ^12^ Even though crystalline silica is hazardous, it is also of great economic importance because it is a valuable raw material for many industrial and manufacturing processes. Precision casting, fiber‐optic cables, raw material for computer chips, petroleum extraction are some of the uses of crystalline silica.^13^

Silica dust is a major environmental and health issue in many quarries^14^ especially in developing countries including Ghana. A study by Scarselli et al,^15^ revealed that exposure to crystalline silica is most common in quarries, construction, and the mining industry. Quarry operations that are sources of dust include drilling, cutting, crushing, breaking, blasting, grinding, and loading. Quarry workers' distance away from the crusher (main dust source) and the use of required PPEs are important in determining the magnitude of dust exposure. Crushing is the most significant dust source in stone quarries and dust concentration decreases with increasing distance away from the crusher.^16^, ^17^, ^18^ Quarry workers are faced with varying concentrations of silica dust depending on the working location. A high degree of respiratory morbidity and eye problems are associated with the stone quarry industry due to the dusty nature of quarry environments. The adverse health effects of working in the stone quarry industry have been well documented.^4^, ^19^, ^20^, ^21^, ^22^, ^23^ Some of the adverse health outcomes mostly experienced by stone quarry workers include eye irritation, breathing difficulty, coughing, and common cold. One way to protect oneself against the risks associated with silica dust exposure is by using the required protective equipment during quarrying operations.

All silica‐related diseases are preventable when the appropriate exposure control measures are applied. Protecting workers from silica dust exposure can be achieved through various exposure control measures.^5^ This can be achieved through eliminating tasks that expose workers to silica dust, substituting crystalline silica materials with non‐crystalline silica materials, using engineering controls such as water spray or local exhaust ventilation, limiting workers access to areas of high RCS concentration and using PPE.^24^ When an organization exhibits good safety culture, it reflects in the safety attitude of the workers. In such working environments, workers are less likely to take health and safety risks. However, workers who find themselves in organizations that exhibit poor organizational safety culture are more likely to have a careless attitude toward health and safety. While it is the responsibility of organizations to provide safe and healthy working environments, it is also the responsibility of workers to follow the laid down safety rules and procedures of the organization to avoid accidents, injuries and work‐related diseases.

Many empirical studies have assessed the health implications of silica dust exposure and the use of required PEE separately. Sairanen and Rinne^17^ observed that dust concentration in stone quarries decreases with increasing distance from the main dust source. The study also mentioned that variation in dust concentration is high within distances ranging from 10 to 200 m. Arcury et al,^25^ and Reed et al,^26^ posited that wearing job‐appropriate PPE is important for decreasing high rates of occupational injury, accidents, and diseases among workers. Even though existing studies have looked at these phenomena separately, there exists a critical knowledge gap in understanding the joint effect of work‐post and PPE usage on self‐reported disease symptoms of stone quarry workers. Knowledge about the joint effect of work‐post and PPE usage is important for developing strategies relevant for addressing the causes of silica‐dust‐related disease symptoms and diseases among quarry workers. In this study, we assessed the joint effect of work‐post (working distance to the crusher) and PPE usage on self‐reported disease symptoms of stone quarry workers while controlling for relevant compositional contextual factors.

## 2 MATERIALS AND METHODS

### 2.1 Study area

**Figure 1:**
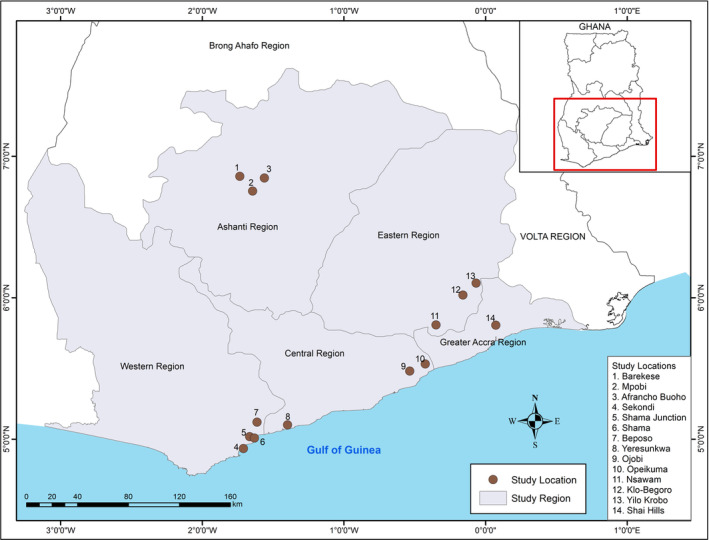
A map showing the communities in which the quarry sites are located in the five selected regions

The study was carried out in the southern part of Ghana where most of the stone quarry sites in the country are located. The five regions selected for this study are noted for quarry activities among the eight regions in southern Ghana. With the exception of Greater Accra Region where we selected six quarry sites from one community (Shai Hills) for data collection, all the other four regions had three communities selected with two sites in each of the community—Central (Yeresunkwa, Ojobi, Opeikuma), Western (Sekondi, Shama, Beposo), Eastern (Nsawam, Klo‐Begoro, Yilo Krobo), and Ashanti (Barekese, Mpobi, Afrancho Buoho). The geographical locations of the study sites are presented in Figure 1.

### 2.2 Data collection and sampling procedure

This study is part of a research project that assessed the human health risks of occupational exposure to silica dust by stone quarry workers in Ghana. In all, 30 stone quarries were visited in the five regions for data collection from May 2018 to February 2019. A total of 600 questionnaires were administered to 20 workers in each quarry site however, 524 filled questionnaires were retrieved from the participants. Even though all participants agreed to take part in the study, 76 did not return their questionnaires. Participants were recruited through a random sampling method. In each quarry site, workers were assigned numbers and a random number generator was used to select 20 workers. Participants of the study were 18 years and above. Before the commencement of the study, the survey instrument was subjected to content validity test to ensure that it included all the items that are essential and eliminates undesirable items. The judgmental approach was used to establish content validity through literature reviews and evaluations by experts. A pretest was conducted on a smaller sample (60 respondents) to identify errors associated with the survey instrument and also assess response latency. Internal consistency of the survey instrument was assessed using cronbach alpha before the actual survey was conducted. After the survey, follow up visits were made to one quarry site in each region to assess the reliability of the responses.

All the procedures carried out in the study were in accordance with the ethical standards of the Minerals Commission of Ghana. A prior permission was obtained from the management of the quarries visited, the aim and details of the study were also explained to them. Oral and written consent was obtained from all the participants before the study. Participants were neither coerced nor financially induced to take part in the study, we explained that their participation was voluntary. They were also informed that the information provided will contribute to the improvement of safety culture in the stone quarry industry in Ghana.

## 3 MEASURES

### 3.1 Response variables

The dependent variables considered in this study were eye irritation, breathing difficulty, coughing, and common cold. For each disease symptom, respondents were asked if they often experience any of the symptoms since they started working in the stone quarry industry. The dichotomous response was coded as 0 (for no) and 1 (for yes).

### 3.2 Key predictor variable

The key independent/explanatory variable was selected based on literature, parsimony, practical significance, and theoretical relevance. The key explanatory variable was derived from combining two variables (working distance to the crusher and wearing of required PPE). This produced the predictor variable called “work‐post PPE usage” with four mutually exclusive groups: 1‐100 m without PPE, 1‐100 m with PPE, above 100 m without PPE, and above 100 m with PPE. Sairanen and Rinne^17^ in their study on dust emission from crushing of hard rock aggregates concluded that variation in dust concentration is high within distances ranging from 10 to 200 m from the main dust source. They also observed in their study that crushing is the most significant dust source in stone quarries. Based on literature, we considered individuals who work within high dust concentration distances (10‐200 m) for the study. The study considered individuals who work between 1 and 100 m from the main dust source and workers who work beyond 100 m from the main dust source.

### 3.3 Compositional and contextual factors

Compositional factors refer to biosocial and socio‐cultural characteristics of the stone quarry workers. Biosocial factors include age, sex, race, and ethnicity while socio‐cultural factors include marital status, income, education, occupation, and religion among others.^27^, ^28^ Contextual factors are location‐specific opportunities in a region or a place.^29^ In this study, the compositional factors included age (young adult: less than 35 years, middle‐aged adult: 35‐55 years), education (no formal education, secondary/higher), household size (small: 1‐5, medium: 6‐10), family status (head, member), marital status (single, married, divorced). The contextual factor was region of residence (Central, Western, Greater Accra, Eastern, and Ashanti).

### 3.4 Data analyses

The data were subjected to univariate and multivariate statistical analyses to examine the relationships and proportions between factors that influence self‐reported disease symptoms while controlling for theoretically relevant compositional and contextual factors. All statistical analyses were performed using Stata 14 (StataCorp, College Station, Texas) SE software.

### 3.5 Univariate analyses

Univariate analyses of predictors of self‐reported disease symptoms were carried out using Pearson chi‐square and Cramer's V statistic. Pearson chi‐square was used to test the association between categorical variables. It is used to estimate if two or more groups of samples are independent or not. Cramer's V statistics assess the strength of association among categorical variables.^30^ The univariate results output was presented as a contingency table. Statistical significance of 0.05 was set for all analyses with a confidence interval of 95%.

### 3.6 Multivariate analyses

The relationship between self‐reported disease symptoms and work‐post PPE usage was determined using complementary log‐log regression models and reported as exponentiated coefficients or odds ratios (OR). An OR of 1 means that the predictor does not affect the odds of reporting a specific disease symptom; OR >1 means that the predictor is associated with higher odds of reporting a specific disease symptom; and OR <1 implies that the predictor is associated with lower odds of reporting a specific disease symptom. Compositional (age, ethnicity, education, household size, family status, marital status, religion) and contextual variable (region of residence) that have been suggested in literature to affect self‐reported disease symptoms were controlled for in the models.

There are a number of model options under the assumption of a binary response (no = 0, yes = 1) to each disease symptom: logit model, probit model, complementary log‐log model, and negative log‐log model depending on the link function of the GLM. Unlike logit and probit, the complementary log‐log function is asymmetrical. The link function of this model is suitable for binary outcomes that are asymmetrical. Complementary log‐log model takes into account the fact that affirmative responses are more probable and gives a better representation. The model was used for the analyses of the relationship between the odds of reporting headache, eye irritation, breathing difficulty, coughing, common cold, and theoretically relevant variables because 55% or more of the responses were affirmative, satisfying the assumption for the model.^31^ Eye irritation (42% no, 58% yes), breathing difficulty (26% no, 74% yes), coughing (19% no, 81% yes), and common cold (20% no, 80% yes). The regression models used in this study are built under the assumption of independence of subjects, but the cross‐sectional survey has a hierarchical structure with respondents nested within the survey clusters, which could potentially bias the standard errors (SE).^32^, ^33^, ^34^

## 4 RESULTS

### 4.1 Descriptive analyses

**Figure 2:**
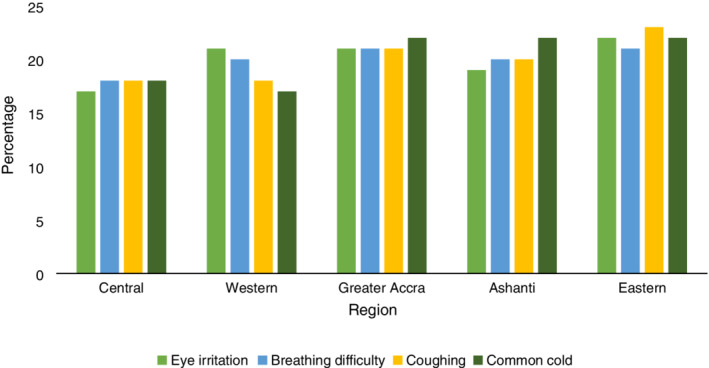
Self‐reported disease symptoms across study regions

Self‐reported disease symptoms across the study regions are shown in Figure 2. A large number of stone quarry workers in this study have experienced disease symptoms associated with silica dust exposure (eye irritation, breathing difficulty, coughing, and common cold) at one point during their working time in the stone quarry sector. Eastern region recorded the highest (22%) likelihood of experiencing eye irritation while Central region recorded the lowest (17%).

Breathing difficulty was highest among quarry workers from Greater Accra and Eastern regions with both regions recording 21%. Reported cases of coughing was highest (23%) in the Eastern region. Common cold was also investigated in this study. Results from Figure 2 shows that common cold was highest in three regions; regions; Greater Accra, Ashanti, and Eastern with all recording 22%. The descriptive statistics suggest that there is a growing number of stone quarry workers in Ghana suffering from silica‐dust‐related disease symptoms which has dire health implications.

**Figure 3:**
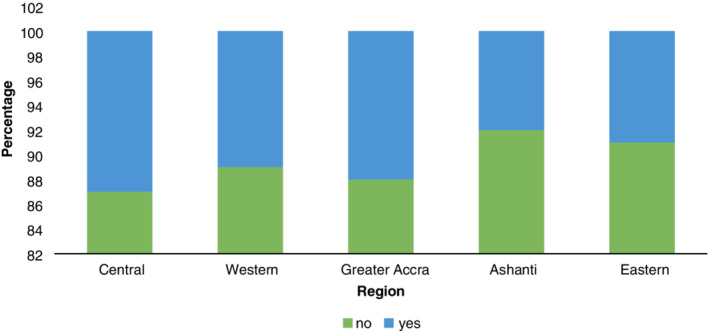
Wearing of personal protective equipment across study regions

Figure 3 shows the percentage of stone quarry workers who were committed to wearing the required PPE at the workplace. The descriptive results revealed that majority of stone quarry workers in Ghana do not wear PPE at work. This is a worrying development due to the risk associated with silica dust exposure. All the study regions performed poorly when it came to workers commitment to wearing the required PPE for quarrying activities. The region that had the worst record was Ashanti, 92% of the workers did not wear PPE. The proportion of workers who did not wear PPE during quarrying in the other regions is as follows; Eastern (91%), Western (89%), Greater Accra (88%), and Central (87%). These outcomes show that there is a huge safety culture gap particularly on the use of PPE by workers in the stone quarry sector.

### 4.2 Univariate analyses

Results for the univariate analyses are provided in Table S1. The Pearson chi‐square statistic rejected the hypothesis that work‐post‐PPE usage is independent of self‐reported disease symptoms. This means that there is a relationship between work‐post PPE usage and reported disease symptoms. Cramer's V statistics (Table S1) show a strong relationship between work‐post PPE usage and self‐reported disease symptoms. The results (Table S1) revealed a weak relationship between age and eye irritation, region also had a weak association with common cold. All the other covariates had no association with any of the self‐reported disease symptoms.

The strength of the association between disease symptoms and work‐post PPE usage based on Cramer's V statistic in decreasing order of magnitude, are as follows: coughing > common cold > breathing difficulty > eye irritation.

### 4.3 Multivariate analyses

Three models were developed at the multivariate level for eye irritation, breathing difficulty, coughing, and common cold; work‐post PPE usage and biosocial factors (model 1), socio‐cultural factors (model 2), and contextual factors (model 3) to assess how they cumulatively influenced exposure to silica dust‐related air pollutants and disease symptoms. Results for models 1 and 2 are provided in the Table S2‐S5 and results for model 3 are presented in Table 1.

**Table 1:** Multivariate complementary log‐log regression model predicting the experience of disease symptoms by stone quarry workers

	Eye Irritation					Breathing Difficulty					Coughing					Common Cold				
Variable	OR	SE	P value	Conf.	Interval	OR	SE	P value	Conf.	Interval	OR	SE	P value	Conf.	Interval	OR	SE	P value	Conf.	Interval
**Work‐post PPE usage (ref: 1‐100m No)**																				
1‐100m Yes	0.100	0.060	**0.000**	0.031	0.324	0.061	0.036	**0.000**	0.019	0.192	0.030	0.020	**0.000**	0.008	0.108	0.072	0.035	**0.000**	0.028	0.185
Above 100m No	1.081	0.137	0.542	0.842	1.386	1.031	0.123	0.796	0.817	1.302	0.836	0.107	0.163	0.650	1.075	0.874	0.104	0.257	0.693	1.103
Above 100m Yes	0.132	0.078	**0.001**	0.042	0.417	0.051	0.036	**0.000**	0.012	0.207	0.014	0.014	**0.000**	0.002	0.097	0.034	0.024	**0.000**	0.008	0.137
**Age group (ref: Young adult)**																				
Middle‐aged Adult	0.523	0.083	**0.000**	0.382	0.715	0.791	0.123	0.132	0.583	1.073	0.527	0.076	**0.000**	0.397	0.699	0.811	0.138	0.219	0.581	1.132
**Educational attainment (ref: No education)**																				
Secondary or higher	0.916	0.120	0.503	0.708	1.184	0.872	0.108	0.268	0.684	1.111	0.827	0.108	0.145	0.641	1.067	0.845	0.109	0.193	0.656	1.089
**Household size (ref: Small)**																				
Medium	0.972	0.129	0.829	0.750	1.260	0.973	0.119	0.822	0.766	1.236	0.896	0.118	0.407	0.692	1.161	0.941	0.120	0.634	0.733	1.209
**Family status (ref: Head)**																				
Member	0.266	0.218	0.107	0.053	1.328	0.605	0.354	0.391	0.192	1.906	0.667	0.452	0.550	0.177	2.518	2.535	1.429	0.099	0.840	7.651
**Marital status (ref: Single)**																				
Married	0.399	0.323	0.257	0.081	1.954	0.539	0.318	0.296	0.170	1.715	0.970	0.662	0.965	0.255	3.695	2.166	1.221	0.170	0.718	6.538
Divorced	0.349	0.305	0.229	0.063	1.939	0.497	0.328	0.289	0.137	1.809	2.141	1.549	0.293	0.519	8.838	3.193	2.019	0.066	0.924	11.026
**Region (ref: Central)**																				
Western	1.399	0.281	0.095	0.944	2.073	1.085	0.208	0.671	0.745	1.581	0.734	0.144	0.115	0.499	1.079	0.813	0.154	0.273	0.561	1.177
Greater Accra	1.154	0.231	0.474	0.779	1.710	1.078	0.196	0.681	0.755	1.538	1.000	0.196	0.999	0.681	1.468	1.155	0.213	0.435	0.804	1.658
Ashanti	1.134	0.230	0.535	0.762	1.689	1.110	0.209	0.581	0.767	1.605	1.141	0.229	0.510	0.770	1.693	1.864	0.394	**0.003**	1.232	2.821
Eastern	1.164	0.243	0.466	0.773	1.753	1.026	0.193	0.891	0.709	1.484	1.368	0.296	0.147	0.895	2.089	1.388	0.266	0.087	0.953	2.021

The P‐values that are significant (<0.05) are the bold values.

### 4.4 Relationship between work‐post PPE usage and eye irritation

Under biosocial factors (Table S2), workers who work between 1 and 100 m from the crusher with the required PPE were 90% less likely to report eye irritation. Quarry workers who work beyond 100 m from the crusher with the required PPE were 87% less likely to report eye irritation compared with their counterparts who work between 1 and 100 m from the crusher without the required PPE. Middle‐aged adults were 37% less likely to report eye irritation compared with the young adult group.

When socio‐cultural factors were controlled for in model 2 (Table S2), the relationship between work‐post PPE usage and experience of eye irritation remained statistically significant. Workers who work between 1 and 100 m and beyond 100 m from the crusher with the required PPE were 90% and 87% less likely to report eye irritation respectively compared with those who work between 1 and 100 m from the crusher without the required PPE. The direction and magnitude of the odds of reporting eye irritation persisted when age was controlled for in this model. Middle‐aged adults were 49% less likely to report eye irritation compared with those in the young‐adults group. However, educational attainment, household size, family status, and marital status were not significant predictors of eye irritation.

Just like it was observed in the biosocial and socio‐cultural models, the relationship between work‐post PPE usage and experience of eye irritation remained robust in the third model (Table 1) in which contextual factors were added. Workers who work between 1‐100 m and beyond 100 m from the crusher with the required PPE were again 90% and 87% respectively less likely to report eye irritation compared with their counterparts who work between 1 and 100 m without the required PPE. The relationship between age and eye irritation persisted under this model following the previous trends. Middle‐aged adults were 48% less likely to report eye irritation compared with young adult workers. Again, educational attainment, household size, family status, marital status, and region did not predict eye irritation in the socio‐cultural model.

### 4.5 Relationship between work‐post PPE usage and breathing difficulty

In model 1(Table S3), stone quarry workers who work between 1‐100 m and beyond 100 m from the crusher with the required PPE were 94% and 95% respectively less likely to report breathing difficulty compared with individuals who work between 1 and 100 m from the crusher without the required PPE. Individuals in the middle‐aged adult category were 26% less likely to report breathing difficulty compared with the young adult group.

Model 2 controlled for socio‐cultural factors (Table S3). The relationship between work‐post PPE usage and experience of breathing difficulty remained robust under this model. Workers who work between 1 and 100 m and beyond 100 m from the crusher with the required PPE were 94% and 95% less likely to report breathing difficulty compared with those who work between 1 and 100 m from the crusher without the required PPE. Unlike the biosocial model where age predicted breathing difficulty, the relationship was not statistically significant in this model. Also, educational attainment, household size, family status, and marital status did not predict breathing difficulty in this model.

The odds observed in model 2 for the relationship between work‐post PPE usage and the likelihood of reporting breathing difficulty persisted in model 3 (contextual model; Table 1). Individuals who work between 1 and 100 m and those who work beyond 100 m from the crusher with the required PPE were 94% and 95% respectively less likely to report breathing difficulty compared with their counterparts who work between 1 and 100 m from the crusher without the required PPE. However, age, educational attainment, household size, family status, marital status, and region did not predict breathing difficulty under this model.

### 4.6 Relationship between work‐post PPE usage and coughing

After controlling for biosocial factors in model 1 (Table S4), the results show that stone quarry workers who work between 1‐100 m and beyond 100 m from the crusher with the required PPE were 96% and 98% respectively less likely to report coughing compared with those who work between 1 and 100 m from the crusher without the required PPE. However, the relationship between age and coughing was not statistically significant in this model.

When socio‐cultural factors were control for in model 2 (Table S4), the relationship between stone quarry workers and the likelihood of reporting coughing was still robust. Respondents who work between 1‐100 m and beyond 100 m from the crusher with the required PPE were 97% and 99% respectively less likely to report coughing compared with individuals who work between 1 and 100 m from the crusher without the required PPE. Unlike in the biosocial model, age significantly predicted coughing in the socio‐cultural model. Individuals in the middle‐aged category were 46% less likely to report coughing compared with their counterparts in the young adult group. Educational attainment, household size, family status, and marital status were not significant predictors of coughing in the socio‐cultural model.

Geographical region was controlled for in model 3 (Table 1). Stone quarry workers who work between 1‐100 m and beyond 100 m from the crusher with the required PPE were 97% and 99% respectively less likely to report coughing compared with the reference group. The relationship between age and coughing remained robust and persisted under this model. Middle‐aged adults were 47% less likely to report coughing compared with young adult workers. However, educational attainment, household size, family status, marital status, and region were not significant predictors of coughing under the contextual model.

### 4.7 Relationship between work‐post PPE usage and common cold

Bio‐social factors were controlled for in model 1 (Table S5). The results revealed that stone quarry workers who work between 1‐100 m and beyond 100 m from the crusher with the required PPE were 92% and 96% respectively less likely to report common cold compared with those who work between 1 and 100 m from the crusher without the required PPE. Age was not a significant predictor of common cold under this model.

When socio‐cultural factors were accounted for in model 2 (Table S5), the relationship between work‐post PPE usage and common cold remained robust and persisted in this model. Stone quarry workers who work between 1‐100 m and beyond 100 m from the crusher with the required PPEs were 93% and 96% respectively less likely to report common cold compared with those who work between 1 and 100 m from the crusher without the required PPE. Just like in the bio‐social model age was not a significant predictor of common cold in this model. Other variables that did not predict common cold under this model were educational attainment, household size, family status, and marital status.

Contextual factor (region) was controlled for in model 3 (Table 1). Just like in the socio‐cultural model, the relationship between work‐post PPE usage and common cold remained robust in this model. Stone quarry workers who work between 1‐100 m and beyond 100 m from the crusher with the required PPEs were 93% and 97% respectively less likely to report common cold compared with their counterparts who work between 1 and 100 m from the crusher without the required PPE. Following the trend from the biosocial and socio‐cultural models, age, educational attainment, household size, family status, and marital status were not significant predictors of common cold. Except for Ashanti region, there was no statistically significant relationship between the four other regions (Central, Western, Eastern, and Greater Accra) and the likelihood of experiencing common cold. Workers in the Ashanti region were 86% more likely to report common compared with their counterparts in the Central region.

## 5 DISCUSSION

This study evaluated the joint effect of work‐post (distance to main dust source—crusher) and the use of required PPE on self‐reported disease symptoms of stone quarry workers in Ghana. Armah et al,^34^ posited that health outcomes can be measured as subjective or perceived health status (self‐rated health). Adverse effects of silica dust exposure on the health of workers in silica‐exposed work environments are a matter of importance, particularly in developing countries like Ghana where workers may be subjected to high exposure levels at industrial sites. Our findings provide strong evidence that short‐term and long‐term silica dust exposure is associated with adverse health outcomes such as eye irritation, breathing difficulty, coughing, and common cold among Ghanaian stone quarry workers. Studies that considered the joint effect of working distance to a stone crusher (where a majority of the silica dust is generated) and the use of required PPE on adverse health outcomes of stone quarry workers in developing countries have generally been unreported. There is a health and safety disadvantage to people who work in silica dust polluted environments. People engaged in stonework, masonry, and construction work are highly exposed to silica dust and are likely to suffer adverse health effects associated with silica dust exposure.^15^ It has been widely reported that occupation influences health outcomes.^4^, ^20^, ^35^, ^36^, ^37^

The findings of this study give a strong indication of the influence of the joint effect of work‐post and PPE usage (work‐post PPE usage) on the likelihood of stone quarry workers experiencing disease symptoms (eye irritation, breathing difficulty, coughing, and common cold). Work‐post PPE usage significantly predicted all the four disease symptoms in all the three models, that is, biosocial, socio‐cultural, and contextual models. Based on our findings, stone quarry workers who protected themselves appropriately with the required PPE and were either close or far away from the crusher (main dust source) are at a lower risk of experiencing any of the disease symptoms (eye irritation, breathing difficulty, coughing, and common cold) than those who did not protect themselves irrespective of the distance from which they worked from the crusher. This finding agrees with the study conducted by Rongo et al,^38^ on occupational exposure and health problems among small‐scale industry workers in Dar es Salaam, Tanzania which found that workers who did not wear PPE and were exposed to dust and fumes reported health complains related to their work. Neves et al,^39^ in their study reported that adherence to PPE usage is determined by individual safety values and beliefs. Several studies have found that stone crushing is the main source of dust in stone quarries.^16^, ^17^, ^18^ This was observed in the quarries visited. Proximity to the crusher only poses a risk to workers who do not use the required PPE. Workers who work further away from the crusher and do not use the required PPE have a higher chance of experiencing adverse health outcomes than those who work closer to the crusher and wear the required PPE. This outcome is an indication that the long‐held perception that the further away one works from the main dust source (crusher) particularly without wearing PPE, the more the person is protected against silica‐dust related ailments is incorrect. It has been reported that respirable silica dust produced by industrial processes poses a potential risk to people working within meters in that environment for years without protection.^13^ These findings give credence to the fact that protecting oneself with PPE against silica dust exposure has huge health and safety benefits.

Our findings also established a significant relationship between age and the likelihood of experiencing eye irritation in the biosocial, socio‐cultural, and contextual models, however, there was no relationship between age and common cold in all three models. The findings show that stone quarry workers who are within the middle‐aged adult group (35‐55 years) have a lower chance of experiencing eye irritation than young adult (below 35 years) workers. This outcome indicates a positive and significant impact of age on the likelihood of stone quarry workers experiencing eye irritation. As the age of the quarry worker increases, the likelihood of experiencing eye irritation decreases. This could be because older workers were more safety conscious and so more committed to protecting their eyes with safety goggles/protective eyewear during quarrying operations than their younger counterparts. This is consistent with a study by Forrest et al,^40^ that reported very few young adults using eye protection compared to their older counterparts while engaging in activities that could cause an eye injury. A study conducted by Lombardi et al,^41^ on factors influencing worker use of personal protective eyewear also found that younger inexperienced workers are less likely to use PPE. Laying more emphasis on eye safety in stone quarries might help increase the use of protective eyewear in this age group. Generally, older workers exhibit a better attitude toward safety and health at work than younger workers. Armah et al,^42^ asserted that older workers are more cautious and less risk‐taking when it comes to safety and health. Reports have shown that young people do not mind working in high‐risk environments because they consider themselves physically healthier and stronger than older workers meanwhile younger workers lack experience and maturity when it comes to work and its associated safety and health risks.^43^ Age also had a significant association with the likelihood of workers experiencing breathing difficulty but only in the biosocial model. The findings show that middle‐aged adults experienced less breathing difficulty than young adults in the biosocial model suggesting that biosocial factors influence the likelihood of experiencing breathing difficulty. However, age did not have any significant relationship with the likelihood of experiencing coughing in the biosocial model but had a significant association in the socio‐cultural and contextual models. This implies that socio‐cultural and contextual factors influence the incidence of coughing among stone quarry workers.

The study found that geographical location (region) has an association with the likelihood of workers experiencing common cold. Workers in the Ashanti region had a higher likelihood of experiencing common cold than those in the Central region. This may be because workers in the Central region were more prudent in using PPE in protecting themselves from the silica dust than those in the Ashanti region. However, the geographical location of the quarries had no statistically significant relationship with the likelihood of workers experiencing eye irritation, breathing difficulty, and coughing. Our findings also revealed that educational attainment, household size, family status, and marital status had no significant association with the four disease symptoms, suggesting that these variables do not influence the likelihood of workers experiencing these disease symptoms.

The results show that majority of quarry workers in Ghana have a high likelihood of experiencing silica‐dust‐related disease symptoms. This is not surprising because majority of the workers in the study regions were not using the prescribed PPE during quarrying and thus exposed to silica dust. Similarly, a study conducted by Singh et al,^44^ on the use of PPE by pesticide applicators in rural India found that majority of the workers were not using protective gear. MacFarlane et al,^45^ also reported non‐use of PPE among Australian grain farmers in his study. Even though these reports were in the agricultural sector, the findings demonstrate the attitude of workers generally toward the use of PPE. Rongo et al,^38^ also reported low use of PPE among small scale industry workers exposed to dust and fumes in Dar es Salaam, Tanzania. The major disease caused by silica dust inhalation is silicosis, a lung disease. Over time, silica dust can build up in the lungs and breathing passages. This leads to inflammation and scarring in the form of nodular lesions in the upper lobes of the lung, making it difficult to breathe. Symptoms of this disease can appear from a few weeks to many years after exposure to silica dust. The main disease symptom of silicosis is difficulty in breathing. Majority of the workers in this study have reported experiencing breathing difficulty. This finding is a major cause for concern especially knowing that silicosis takes years to fully manifest. It is even more worrying because silicosis has no cure. However, with the right safety measures in place, silicosis can be prevented. A key strategy in preventing silicosis is by using job‐appropriate PPE during stone quarrying operations.

### 5.1 Limitations of the study

The key strength of this study is its empirical disposition as respondents were real workplace employees. However, one of the limitations of the study is the reliance on self‐reported measures to assess the likelihood of experiencing silica‐dust‐related disease symptoms. Outcomes of the relationship among the measures may, therefore, be confounded by common method variance. Wagner and Crampton^46^ indicated in their meta‐analytic study that even though this problem continues to be cited regularly, the immensity of the distortions may be exaggerated. It has been well documented in literature that self‐reported measures have proven to be effective for organizational safety studies.^47^ Furthermore, the study design made it impossible for questions relating to the relationship between cause and effect to be included in the questionnaire. Tests of the hypotheses were conducted on self‐reported survey data therefore we could not evaluate the reported symptoms with medical records of respondents. The inclusion of actual medical records of stone quarry workers could have added some vital information to this study. Using stone quarry workers' reports of disease symptoms is only an estimate of disease symptoms suffered by them and this is likely to be subject to recall bias. Notwithstanding the limitations of using self‐reported measures, our findings showed a clear relationship between work‐post PPE usage and the four disease symptoms experienced.

## 6 CONCLUSION

The profile of Ghana's silica dust exposed population is not well studied and documented. Stone quarry workers are at high risk of developing silica‐related symptoms and diseases due to the cumulative effect of silica dust exposure, a finding that is supported by a large body of epidemiologic evidence. This study assessed the joint effect of work‐post (distance to main dust source‐crusher) and PPE usage on the likelihood of workers experiencing adverse health outcomes (eye irritation, breathing difficulty, coughing, and common cold) in stone quarries in Ghana. The study found that stone quarry workers who work between 1‐100 m and beyond 100 m from the crusher with the required PPE reported lower likelihoods of experiencing eye irritation, breathing difficulty, coughing, and common cold. Individuals who work between 1‐100 m and beyond 100 m from the crusher without the required PPE reported a higher likelihood of experiencing adverse health outcomes. This relationship was robust and persisted even when it was subjected to compositional and contextual attributes. Middle‐aged adults reported lower frequencies of adverse health outcomes.

Across the five study regions, we found that majority of workers were not using PPE during quarrying operations. This is a major health and safety risk that can potentially put workers in a vulnerable position of developing the deadly silicosis disease. These findings provide a better understanding of current negative safety practices in the stone quarry sector and may help in the development of programs to promote silica dust exposure control. There are a number of adverse health implications for people who work in silica dust polluted environments, suggesting the need for a national occupational safety and health policy, silica dust control interventions, and health promotion campaigns to target silica‐related occupations. As a key regulatory measure, Ghana needs to come up with a recommended RCS permissible exposure limit for silica‐exposed occupations per international standards.

## AUTHOR CONTRIBUTIONS

Conceptualization: Dzifa Francis Ahadzi

Data Curation: Abdul‐Rahaman Afitiri

Formal Analysis: Dzifa Francis Ahadzi, Abdul‐Rahaman Afitiri, Bernard Ekumah

Investigation: Verona Kanatey, Abdullah Afedzi

Methodology: Dzifa Francis Ahadzi, Abdul‐Rahaman Afitiri, Bernard Ekumah

Project Administration: Verona Kanatey, Abdullah Afedzi

Writing ‐ Original Draft: Dzifa Francis Ahadzi, Abdul‐Rahaman Afitiri

Writing ‐ Review and Editing: Dzifa Francis Ahadzi, Abdul‐Rahaman Afitiri, Bernard Ekumah, Verona Kanatey, Abdullah Afedzi

All authors have read and approved the final version of the manuscript.

Dzifa Francis Ahadzi had full access to all of the data in the study and takes complete responsibility for the integrity of the data and the accuracy of the data analysis.

## ETHICS STATEMENT

The study was conducted in 30 stone quarries in five regions in Ghana (Central, Western, Greater Accra, Eastern, and Ashanti regions). All the procedures carried out in the study were in accordance with the ethical standards of the Minerals Commission and the Environmental Protection Agency of Ghana. Prior permission was obtained from the management of the quarries visited. Participants were not coerced or financially induced to take part in the study. A comprehensive justification of the study procedure, purpose, and benefits were made known to all the participants and their wish to join the study was voluntary and hence could freely opt out during or after the survey period. Oral and written consent was obtained from all the participants before the study. In principle, each participant was required to acknowledge that they fully understood the reason for the study and what was required of them.

## CONFLICT OF INTEREST

The authors declare no conflict of interest.

## TRANSPARENCY STATEMENT

Dzifa Francis Ahadzi affirms that this manuscript is an honest, accurate, and transparent account of the study being reported; that no important aspects of the study have been omitted; and that any discrepancies from the study as planned (and, if relevant, registered) have been explained.

## Supporting information


**Table S1**. Percentage distribution of disease symptoms by predictor variable
**Table S2**. Multivariate complementary log‐log regression model predicting the experience of eye irritation by stone quarry workers
**Table S3**. Multivariate complementary log‐log regression model predicting the experience of breathing difficulty by stone quarry workers
**Table S4**. Multivariate complementary log‐log regression model predicting the experience of coughing by stone quarry workers
**Table S5**. Multivariate complementary log‐log regression model predicting the experience of common cold by stone quarry workers

## Data Availability

Due to the nature of this research, participants of this study did not agree for their data to be shared publicly, so supporting data is not available.
