# Supplementary material for: Self‐reported disease symptoms of stone quarry workers exposed to silica dust in Ghana
Source: Health Sci Rep. 2020 Oct 5;3(4):e189. doi: 10.1002/hsr2.189 (PMC7534518; doi:10.1002/hsr2.189)
Supplement: Supplementary file 1 — Table S1. Percentage distribution of disease symptoms by predictor variable Table S2. Multivariate complementary log‐log regression model predicting the experience of eye irritation by stone quarry workers Table S3. Multivariate complementary log‐log regression model predicting the experience of breathing difficulty by stone quarry workers Table S4. Multivariate complementary log‐log regression model predicting the experience of coughing by stone quarry workers Table S5. Multivariate complementary log‐log regression model predicting the experience of common cold by stone quarry workers [file HSR2-3-e189-s001.docx]

**Appendix**

Table S1: Percentage distribution of disease symptoms by predictor variable

| **Work-post PPE Usage** | | | | | | |
| --- | --- | --- | --- | --- | --- | --- |
| Disease Symptoms | 1-100m No (%) | 1-100m Yes (%) | Above 100m No (%) | Above 100m Yes (%) | Inferential Statistics | |
| Eye irritation |  | | | | | |
| No | 36 | 12 | 42 | 10 | χ^2^ = 54.4234 (P-value = 0.000; Cramér's V = 0.3223) | |
| Yes | 45 | 1 | 53 | 1 |  |  |
| Breathing difficulty |  | | | | | |
| No | 30 | 20 | 34 | 16 | χ^2^= 132.9250 (P-value = 0.000; Cramér's V =0.5037) | |
| Yes | 46 | 1 | 53 | 1 |  |  |
| Coughing |  | | | | | |
| No | 16 | 27 | 33 | 23 | χ^2^ = 216.1469 (P-value = 0.000; Cramér's V = 0.6423) | |
| Yes | 47 | 1 | 52 | 0 |  |  |
| Common cold |  | | | | | |
| No | 23 | 24 | 33 | 21 | χ^2^ = 167.6503 (P-value = 0.000; Cramér's V = 0.5656) | |
| Yes | 46 | 1 | 52 | 0 |  |  |
| **Age** | | | | | | |
| Disease Symptoms | Young Adult (%) | | Middle-aged Adult (%) | | Inferential Statistics | |
| Eye irritation |  | | | | | |
| No | 25.91 |  | 74.09 |  | χ^2^ = 10.4988 (P-value = 0.001; Cramér's V = -0.1415) | |
| Yes | 39.47 |  | 60.53 |  |  |  |
| Breathing difficulty |  | | | | | |
| No | 27.41 |  | 72.59 |  | χ^2^ = 3.3000 (P-value = 0.069; Cramér's V = -0.0794) | |
| Yes | 35.99 |  | 64.01 |  |  |  |
| Coughing |  | | | | | |
| No | 28.28 |  | 71.72 |  | χ^2^ = 1.6482 (P-value = 0.199; Cramér's V = -0.0561) | |
| Yes | 35.06 |  | 64.94 |  |  |  |
| Common cold |  | | | | | |
| No | 30.19 |  | 69.81 |  | χ^2^ = 0.7656 (P-value = 0.382; Cramér's V = -0.0382) | |
| Yes | 34.69 |  | 65.31 |  |  |  |
| **Educational Attainment** | | | | | | |
| Disease Symptoms | No Formal Education (%) | | Secondary/Higher (%) | | Inferential Statistics | |
| Eye irritation |  | | | | | |
| No | 57.73 |  | 42.27 |  | χ^2^ = 0.0014 (P-value = 0.971; Cramér's V = 0.0016) | |
| Yes | 57.57 |  | 42.43 |  |  |  |
| Breathing difficulty |  | | | | | |
| No | 59.26 |  | 40.74 |  | χ^2^ = 0.1968 (P-value = 0.657; Cramér's V = 0.0194) | |
| Yes | 57.07 |  | 42.93 |  |  |  |
| Coughing |  | | | | | |
| No | 57.58 |  | 42.42 |  | χ^2^ = 0.0002 (P-value = 0.990; Cramér's V = -0.0006) | |
| Yes | 57.63 |  | 42.37 |  |  |  |
| Common cold |  | | | | | |
| No | 60.38 |  | 39.62 |  | χ^2^ = 0.4097 (P-value = 0.522; Cramér's V = 0.0280) | |
| Yes | 56.94 |  | 43.06 |  |  |  |
| **Household Size** | | | | | | |
| Disease Symptoms | Small (%) | | Medium (%) | | Inferential Statistics | |
| Eye irritation |  | | | | | |
| No | 60.91 |  | 39.09 |  | χ^2^ = 0.6956 (P-value = 0.404; Cramér's V = -0.0364) | |
| Yes | 64.47 |  | 35.53 |  |  |  |
| Breathing difficulty |  | | | | | |
| No | 61.48 |  | 38.52 |  | χ^2^ = 0.1745 (P-value = 0.676; Cramér's V = -0.0182) | |
| Yes | 63.50 |  | 36.50 |  |  |  |
| Coughing |  | | | | | |
| No | 61.62 |  | 38.38 |  | χ^2^ = 0.0970 (P-value = 0.756; Cramér's V = -0.0136) | |
| Yes | 63.29 |  | 36.71 |  |  |  |
| Common cold |  | | | | | |
| No | 61.32 |  | 38.68 |  | χ^2^ = 0.1564 (P-value = 0.693; Cramér's V = -0.0173) | |
| Yes | 63.40 |  | 36.60 |  |  |  |
| **Family Status** | | | | | | |
| Disease Symptoms | Head (%) | | Member (%) | | Inferential Statistics | |
| Eye irritation |  | | | | | |
| No | 78.18 |  | 21.82 |  | χ^2^ = 0.0224 (P-value = 0.881; Cramér's V = 0.0065) | |
| Yes | 77.63 |  | 22.37 |  |  |  |
| Breathing difficulty |  | | | | | |
| No | 82.22 |  | 17.78 |  | χ^2^ = 2.0052 (P-value = 0.157; Cramér's V = 0.0619) | |
| Yes | 76.35 |  | 23.65 |  |  |  |
| Coughing |  | | | | | |
| No | 76.77 |  | 23.23 |  | χ^2^ = 0.0849 (P-value = 0.771; Cramér's V = -0.0127) | |
| Yes | 78.12 |  | 21.88 |  |  |  |
| Common cold |  | | | | | |
| No | 81.13 |  | 18.87 |  | χ^2^ = 0.8241 (P-value = 0.364; Cramér's V = 0.0397) | |
| Yes | 77.03 |  | 22.97 |  |  |  |
| **Marital Status** | | | | | | |
| Disease Symptoms | Single (%) | Married (%) | Divorced (%) | | Inferential Statistics | |
| Eye irritation |  | | | | | |
| No | 20.45 | 73.64 | 5.91 |  | χ^2^ = 1.3260 (P-value = 0.515; Cramér's V = 0.0503) | |
| Yes | 22.70 | 73.35 | 3.95 |  |  |  |
| Breathing difficulty |  | | | | | |
| No | 16.30 | 76.30 | 7.41 |  | χ^2^ = 5.3379 (P-value = 0.069; Cramér's V = 0.1009) | |
| Yes | 23.65 | 72.49 | 3.86 |  |  |  |
| Coughing |  | | | | | |
| No | 21.21 | 72.73 | 6.06 |  | χ^2^ = 0.4513 (P-value = 0.798; Cramér's V = 0.0293) | |
| Yes | 21.88 | 73.65 | 4.47 |  |  |  |
| Common cold |  | | | | | |
| No | 18.87 | 74.53 | 6.60 |  | χ^2^ = 1.4651 (P-value = 0.481; Cramér's V = 0.0529) | |
| Yes | 22.49 | 73.21 | 4.30 |  |  |  |
| **Region** | | | | | | |
| Disease Symptoms | Central (%) | Western (%) | Greater Accra (%) | Ashanti (%) | Eastern (%) | Inferential statistics |
| Eye irritation |  | | | | | |
| No | 22.73 | 16.82 | 21.82 | 20.00 | 18.64 | χ^2^ = 4.7942 (P-value = 0.309; Cramér's V = 0.0957) |
| Yes | 16.45 | 21.05 | 21.38 | 18.75 | 22.37 |  |
| Breathing difficulty |  | | | | | |
| No | 22.22 | 17.78 | 22.22 | 17.78 | 20.00 | χ^2^ = 1.4540 (P-value = 0.835; Cramér's V = 0.0527) |
| Yes | 17.99 | 19.79 | 21.34 | 19.79 | 21.08 |  |
| Coughing |  | | | | | |
| No | 23.23 | 26.26 | 22.22 | 16.16 | 12.12 | χ^2^ = 9.2525 (P-value = 0.055; Cramér's V = 0.1329) |
| Yes | 18.12 | 17.65 | 21.41 | 20.00 | 22.82 |  |
| Common cold |  | | | | | |
| No | 24.53 | 27.36 | 21.70 | 10.38 | 16.04 | χ^2^ = 13.4772 (P-value = 0.009; Cramér's V = 0.1604) |
| Yes | 17.70 | 17.22 | 21.53 | 21.53 | 22.01 |  |

Table S2: Multivariate complementary log-log regression model predicting the experience of eye irritation by stone quarry workers

| **Variable** | **Work-post PPE usage + Biosocial factors** | | | | | **+ Socio-cultural factors** | | | | |
| --- | --- | --- | --- | --- | --- | --- | --- | --- | --- | --- |
|  | **OR** | **SE** | **P value** | **Conf.** | **Interval** | **OR** | **SE** | **P value** | **Conf.** | **Interval** |
|  | **Model 1** | | | | | **Model 2** | | | | |
| **Work-post PPE usage (ref: 1-100m No)** |  |  |  |  |  |  |  |  |  |  |
| 1-100m Yes | 0.101 | 0.06 | **0.000** | 0.031 | 0.322 | 0.102 | 0.061 | **0.000** | 0.031 | 0.329 |
| Above 100m No | 1.018 | 0.124 | 0.881 | 0.802 | 1.294 | 1.074 | 0.136 | 0.572 | 0.839 | 1.375 |
| Above 100m Yes | 0.132 | 0.077 | **0.000** | 0.042 | 0.413 | 0.131 | 0.076 | **0.000** | 0.042 | 0.409 |
| **Age group (ref: Young adult)** |  |  |  |  |  |  |  |  |  |  |
| Middle-aged Adult | 0.633 | 0.079 | **0.000** | 0.496 | 0.808 | 0.514 | 0.081 | **0.000** | 0.377 | 0.699 |
| **Education (ref: No education)** |  |  |  |  |  |  |  |  |  |  |
| Secondary or higher |  |  |  |  |  | 0.922 | 0.117 | 0.524 | 0.719 | 1.183 |
| **Household size (ref: Small)** |  |  |  |  |  |  |  |  |  |  |
| Medium |  |  |  |  |  | 0.963 | 0.125 | 0.769 | 0.747 | 1.241 |
| **Family status (ref: Head)** |  |  |  |  |  |  |  |  |  |  |
| Member |  |  |  |  |  | 0.281 | 0.231 | 0.122 | 0.056 | 1.405 |
| **Marital status (ref: Single)** |  |  |  |  |  |  |  |  |  |  |
| Married |  |  |  |  |  | 0.417 | 0.339 | 0.282 | 0.085 | 2.055 |
| Divorced |  |  |  |  |  | 0.378 | 0.331 | 0.266 | 0.068 | 2.103 |

Table S3. Multivariate complementary log-log regression model predicting the experience of breathing difficulty by stone quarry workers

| **Variable** | **Work-post PPE usage + Biosocial factors** | | | | | **+ Socio-cultural factors** | | | | |
| --- | --- | --- | --- | --- | --- | --- | --- | --- | --- | --- |
|  | **OR** | **SE** | **P value** | **Conf.** | **Interval** | **OR** | **SE** | **P value** | **Conf.** | **Interval** |
|  | **Model 1** | | | | | **Model 2** | | | | |
| **Work-post PPE usage (ref: 1-100m No)** |  |  |  |  |  |  |  |  |  |  |
| 1-100m Yes | 0.060 | 0.035 | **0.000** | 0.019 | 0.191 | 0.061 | 0.036 | **0.000** | 0.019 | 0.192 |
| Above 100m No | 1.005 | 0.116 | 0.965 | 0.801 | 1.261 | 1.028 | 0.123 | 0.814 | 0.814 | 1.300 |
| Above 100m Yes | 0.050 | 0.036 | **0.000** | 0.012 | 0.205 | 0.051 | 0.036 | **0.000** | 0.012 | 0.206 |
| **Age group (ref: Young adult)** |  |  |  |  |  |  |  |  |  |  |
| Middle-aged Adult | 0.739 | 0.090 | **0.013** | 0.582 | 0.939 | 0.790 | 0.120 | 0.121 | 0.586 | 1.065 |
| **Education (ref: No education)** |  |  |  |  |  |  |  |  |  |  |
| Secondary or higher |  |  |  |  |  | 0.886 | 0.107 | 0.314 | 0.699 | 1.122 |
| **Household size (ref: Small)** |  |  |  |  |  |  |  |  |  |  |
| Medium |  |  |  |  |  | 0.969 | 0.118 | 0.793 | 0.764 | 1.229 |
| **Family status (ref: Head)** |  |  |  |  |  |  |  |  |  |  |
| Member |  |  |  |  |  | 0.615 | 0.357 | 0.402 | 0.197 | 1.918 |
| **Marital status (ref: Single)** |  |  |  |  |  |  |  |  |  |  |
| Married |  |  |  |  |  | 0.549 | 0.323 | 0.309 | 0.173 | 1.740 |
| Divorced |  |  |  |  |  | 0.506 | 0.332 | 0.299 | 0.140 | 1.831 |

Table S4. Multivariate complementary log-log regression model predicting the experience of coughing by stone quarry workers

| **Variable** | **Work-post PPE usage + Biosocial factors** | | | | | **+ Socio-cultural factors** | | | | |
| --- | --- | --- | --- | --- | --- | --- | --- | --- | --- | --- |
|  | **OR** | **SE** | **P value** | **Conf.** | **Interval** | **OR** | **SE** | **P value** | **Conf.** | **Interval** |
|  | **Model 1** | | | | | **Model 2** | | | | |
| **Work-post PPE usage (ref: 1-100m No)** |  |  |  |  |  |  |  |  |  |  |
| 1-100m Yes | 0.039 | 0.023 | **0.000** | 0.012 | 0.124 | 0.031 | 0.020 | **0.000** | 0.009 | 0.108 |
| Above 100m No | 0.778 | 0.096 | 0.050 | 0.611 | 0.989 | 0.841 | 0.106 | 0.167 | 0.657 | 1.075 |
| Above 100m Yes | 0.016 | 0.016 | **0.000** | 0.002 | 0.114 | 0.014 | 0.014 | **0.000** | 0.002 | 0.103 |
| **Age group (ref: Young adult)** |  |  |  |  |  |  |  |  |  |  |
| Middle-aged Adult | 0.781 | 0.103 | 0.060 | 0.604 | 1.010 | 0.540 | 0.071 | **0.000** | 0.417 | 0.700 |
| **Education (ref: No education)** |  |  |  |  |  |  |  |  |  |  |
| Secondary or higher |  |  |  |  |  | 0.815 | 0.101 | 0.098 | 0.640 | 1.038 |
| **Household size (ref: Small)** |  |  |  |  |  |  |  |  |  |  |
| Medium |  |  |  |  |  | 0.947 | 0.123 | 0.677 | 0.734 | 1.222 |
| **Family status (ref: Head)** |  |  |  |  |  |  |  |  |  |  |
| Member |  |  |  |  |  | 0.519 | 0.335 | 0.309 | 0.147 | 1.836 |
| **Marital status (ref: Single)** |  |  |  |  |  |  |  |  |  |  |
| Married |  |  |  |  |  | 0.828 | 0.538 | 0.771 | 0.232 | 2.956 |
| Divorced |  |  |  |  |  | 1.705 | 1.168 | 0.436 | 0.445 | 6.528 |

Table S5. Multivariate complementary log-log regression model predicting the experience of common cold by stone quarry workers

| **Variable** | **Work-post PPE usage + Biosocial factors** | | | | | **+ Socio-cultural factors** | | | | |
| --- | --- | --- | --- | --- | --- | --- | --- | --- | --- | --- |
|  | **OR** | **SE** | **P value** | **Conf.** | **Interval** | **OR** | **SE** | **P value** | **Conf.** | **Interval** |
|  | **Model 1** | | | | | **Model 2** | | | | |
| **Work-post PPE usage (ref: 1-100m No)** |  |  |  |  |  |  |  |  |  |  |
| 1-100m Yes | 0.080 | 0.037 | **0.000** | 0.032 | 0.199 | 0.073 | 0.035 | **0.000** | 0.029 | 0.186 |
| Above 100m No | 0.887 | 0.104 | 0.307 | 0.705 | 1.117 | 0.892 | 0.104 | 0.327 | 0.709 | 1.122 |
| Above 100m Yes | 0.039 | 0.028 | **0.000** | 0.010 | 0.157 | 0.038 | 0.027 | **0.000** | 0.009 | 0.153 |
| **Age group (ref: Young adult)** |  |  |  |  |  |  |  |  |  |  |
| Middle-aged Adult | 0.818 | 0.101 | 0.102 | 0.642 | 1.041 | 0.789 | 0.123 | 0.128 | 0.581 | 1.071 |
| **Education (ref: No education)** |  |  |  |  |  |  |  |  |  |  |
| Secondary or higher |  |  |  |  |  | 0.889 | 0.105 | 0.319 | 0.705 | 1.121 |
| **Household size (ref: Small)** |  |  |  |  |  |  |  |  |  |  |
| Medium |  |  |  |  |  | 0.97 | 0.120 | 0.805 | 0.762 | 1.235 |
| **Family status (ref: Head)** |  |  |  |  |  |  |  |  |  |  |
| Member |  |  |  |  |  | 1.808 | 1.002 | 0.285 | 0.610 | 5.357 |
| **Marital status (ref: Single)** |  |  |  |  |  |  |  |  |  |  |
| Married |  |  |  |  |  | 1.747 | 0.975 | 0.317 | 0.585 | 5.214 |
| Divorced |  |  |  |  |  | 2.287 | 1.446 | 0.191 | 0.662 | 7.900 |
